# Petiolate wings: effects on the leading-edge vortex in flapping flight

**DOI:** 10.1098/rsfs.2016.0084

**Published:** 2017-02-06

**Authors:** Nathan Phillips, Kevin Knowles, Richard J. Bomphrey

**Affiliations:** 1 Structure and Motion Laboratory, Royal Veterinary College, University of London, Hatfield AL9 7TA, UK; 2 Aeromechanical Systems Group, Centre for Defence Engineering, Cranfield University, Defence Academy of the United Kingdom, Shrivenham SN6 8LA, UK

**Keywords:** leading-edge vortex, flapping wing, petiolation, insect flight, micro air vehicle

## Abstract

The wings of many insect species including crane flies and damselflies are petiolate (on stalks), with the wing planform beginning some distance away from the wing hinge, rather than at the hinge. The aerodynamic impact of flapping petiolate wings is relatively unknown, particularly on the formation of the lift-augmenting leading-edge vortex (LEV): a key flow structure exploited by many insects, birds and bats to enhance their lift coefficient. We investigated the aerodynamic implications of petiolation *P* using particle image velocimetry flow field measurements on an array of rectangular wings of aspect ratio 3 and petiolation values of *P* = 1–3. The wings were driven using a mechanical device, the ‘Flapperatus’, to produce highly repeatable insect-like kinematics. The wings maintained a constant Reynolds number of 1400 and dimensionless stroke amplitude Λ* (number of chords traversed by the wingtip) of 6.5 across all test cases. Our results showed that for more petiolate wings the LEV is generally larger, stronger in circulation, and covers a greater area of the wing surface, particularly at the mid-span and inboard locations early in the wing stroke cycle. In each case, the LEV was initially arch-like in form with its outboard end terminating in a focus-sink on the wing surface, before transitioning to become continuous with the tip vortex thereafter. In the second half of the wing stroke, more petiolate wings exhibit a more detached LEV, with detachment initiating at approximately 70% and 50% span for *P* = 1 and 3, respectively. As a consequence, lift coefficients based on the LEV are higher in the first half of the wing stroke for petiolate wings, but more comparable in the second half. Time-averaged LEV lift coefficients show a general rise with petiolation over the range tested.

## 1. Introduction

Insects have long been admired for their remarkable flight capabilities, exhibiting impressive load lifting, efficiency and aerial agility. It is because of these characteristics that there has been great interest in their flight not just in the context of understanding the natural world, but also for application to miniature flapping-wing vehicles, or ‘flappercraft’ (e.g. [1,2]). Insect-like flight with reciprocating wings is fundamentally different from that of fixed-wing aircraft owing to the high-frequency, intricate kinematic patterns that result in unsteady and complex aerodynamics. The flapping cycle is often described as being composed of two translation phases and two rapid rotation phases. The translations are the downstroke and upstroke, where the majority of the lift is produced as the wing sweeps with a relatively constant angle of attack. These are separated by two rotation phases, supination and pronation, where the wing reverses direction and pitches to adjust the angle of attack such that the dorsal or ventral surfaces can each act as the aerodynamic suction surface. The majority of the lift produced originates from an intense leading-edge vortex (LEV) that forms along the wing leading edge during the downstroke and—in many insects—also during the upstroke. The wing angle of attack is too high for attached, steady flow, as is found over a typical fixed-wing aircraft and, instead the flow separates at the sharp leading edge and rolls up into an LEV. Its presence over the wing augments lift by reducing the upper wing surface pressure as a result of the concentrated low pressure in the LEV core.

The LEV was first reported for insect-like flapping wings in experiments by Maxworthy [3], who observed its formation on a mechanical model and also reported the presence of a spanwise flow in the LEV core towards the wingtip. This flow was said to transport vorticity out of the LEV and into the tip vortex, resulting in a stable LEV that remains attached to the wing upper surface rather than shedding into the wake. These observations were later supported in experiments on a mechanical model of a hawkmoth by Ellington *et al.* [4]. Their smoke visualizations revealed a conical LEV structure with a larger diameter towards the wingtip and a spanwise flow comparable to the mean wingtip speed. Further studies on the same model revealed similar observations of a conical LEV that grows in size throughout the wing stroke, but breaks down and detaches at its outboard end in the latter half of the wing downstroke [5,6]. Numerous other studies employing mechanical models have since described further details of the LEV formation and its characteristics [7–9]. Rather than being purely conical in form, the LEV has alternatively been reported to be more cylindrical in structure during hover at low Reynolds numbers [10] and in forward flight for several insect species [8,11,12]. The LEV appears to be a widespread feature of flapping flight as it has been observed on live insects [4,13,14], bats [15], birds [16,17] and even on autorotating maple seeds [18].

In addition to the LEV, many other aspects of insect-like flight remain relatively unexplored and are in need of further study to inform the design and development of future robotic flappercraft. In particular, the effects of various aspects of wing shape, including wing petiolation, have received relatively little attention. Here, petiolation refers to the extent that an insect wing is petiolate (on a petiole or stalk) such that the root end of the wing planform begins some distance from the centre of rotation rather than immediately at the wing hinge. In this work, petiolation ‘*P*’ is defined as the distance from the centre of rotation to the wing root in mean chord lengths 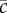 (table 1). Some species, such as crane flies and damselflies, have very petiolate wings with values of *P* = approximately 0.8 and approximately 1.5, respectively. The aerodynamic implications of this parameter and effects on the LEV are not well understood. Shifting the wing planform area further from the wing hinge could have certain predictable benefits. For example, agility could be improved by affording higher manoeuvring torques resulting from a longer moment arm for the centre of pressure. Alternatively, petiolate wings may have no aerodynamic benefits, and may have been selected for other reasons, such as improved clearance of the legs and halteres during flapping.

**Table 1.** Nomenclature.

AR	wing aspect ratio 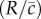
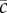	mean wing chord
*C_L_*	lift coefficient
D*	normalized LEV diameter
*f*	flapping frequency
*P*	petiolation 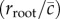
*R*	wing length from root to tip
r*	non-dimensionalized radius
*r*_root_	wing root radius
*r*_tip_	wingtip radius
*Re*	Reynolds number 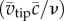
*Ro*	Rossby number 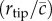
*T*	flapping period (1/*f*)
t*	non-dimensionalized time
*v*_t_	tangential velocity
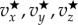	normalized velocity components
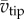	mean wingtip speed
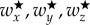	normalized vorticity components
*X_I_*, *Y_I_*, *Z_I_*	inertial coordinate system
*x*, *y*, *z*	rotating coordinate system
*x_w_*, *y_w_*, *z_w_*	wing-fixed coordinate system
*α*	pitch angle
Γ*	normalized LEV circulation
Λ*	dimensionless stroke amplitude
λ*	dimensionless stroke position
*ν*	kinematic viscosity of fluid
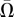	mean wing angular velocity
*ϕ*	stroke angle
*θ*	plunge angle

For the case of a revolving wing with zero, or very little (less than 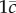) petiolation, the LEV has been shown to be stable and remain attached to the wing [7,19] even throughout continual revolutions [20,21]. In the case of infinite petiolation, however, representing a purely translating wing, the LEV is seen to form and shed within the first few chords of travel from rest [22–25]. After finding that kinematics could stabilize the LEV using computational methods [26], follow-on experimental studies by Lentink & Dickinson [21] explored the effects on the aerodynamic forces and LEV stability for cases of varying Rossby number *Ro*. This dimensionless number describes the ratio of inertial to Coriolis forces, and for convenience is defined as the ratio of the tip radius to the mean wing chord. Thus, increasing the Rossby number is synonymous with increasing petiolation if wing area is kept constant. Their cases ranged from low *Ro* (2.9), to infinite (purely translating wing) and it was concluded that the LEV remains attached to the wing for an *Ro* of 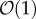. In addition, force measurements revealed that higher Rossby numbers (or petiolation) result in reduced maximum lift coefficients, where those for a translating wing were significantly lower than for a revolving wing. They concluded that, at low *Ro*, Coriolis forces stabilize the LEV and keep it attached. Recent numerical studies exploring the contributions of different fluid forces have supported their result [27].

The effect on force coefficients of increasing petiolation has been explored experimentally by Schlueter *et al.* [28]. Here, petiolation was described as ‘root cutout’ for a range of rectangular, unidirectional revolving wings at a fixed incidence. The authors addressed the problem that the choice of the characteristic velocity is not straightforward when computing lift coefficients. This is because, as petiolation increases, the tangential velocity profile across the span changes proportionally. They formulated axis-relative and root-relative methods to normalize forces according to a defined radius from the centre of rotation or relative to the position from the wing root along the span, respectively. They concluded that for transient cases the root-relative method should be used in comparing force coefficients for varying petiolation, whereas the axis-relative method should be used for steady-state cases.

Further experiments into petiolation effects have been performed on rectangular revolving wings [29,30]. Here, petiolation was varied by changing the wing radius of gyration. Results revealed that in changing from a small to moderate radius of gyration, the LEV topology transitions from a conical to an arch-shaped form [30]. In addition, the outboard portion of the LEV remains comparatively less coherent for the higher petiolation case. Experiments on live insects comparing the downwash profiles of dragonflies (Anisoptera) and damselflies (Zygoptera), with the latter having wings that are more petiolate, found that higher petiolation results in significant upwash in the inboard regions due to stronger root vortices [31]. This results in poorer tip-to-tip span efficiency in contrast with the more even downwash profile of a non-petiolate, tapering wing.

Previous studies comparing the two extremes of a purely revolving (*P* = 0), and purely translating wing (*P* = *∞*) have consistently demonstrated a conical attached LEV, and an arch-shaped detaching LEV for rotation and translation, respectively [32–35]. Recently, it has been shown that between rotation and translation cases, the progression of the LEV over the wing surface is initially very similar before the LEV detaches for the translating wing, but remains attached if the wing is rotating [36]. A large collaborative effort across many research groups compared cases of revolving and translating rectangular wings at both fixed and time-varying angles of attack [37,38]. They found consistent LEV shedding for the translating wings [37] and that, whether wings were revolving or translating, there was no significant effect on the mean force coefficients after the initial vortex growth [38]. This suggests that whether the LEV is attached to a revolving wing, or detaching from a translating wing the lift generated does not differ substantially. This is in contradiction to previous established findings that increasing wing petiolation leads to a decline in lift [21].

## 2. Aims and objectives

Most of the studies into wing petiolation effects have focused on the two extreme cases of revolving and translating wings. The progressive changes in the flow field over more moderate and biologically relevant changes in petiolation within the range of *P* of 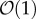 are not as well explored. The majority of the literature describes continually revolving wings at a fixed angle of attack, which is a useful approach in studying isolated elements of the insect flapping cycle, namely mid-downstroke and upstroke. However, in moving towards a complete understanding of insect-like flight, it is important to have a comprehensive approach that includes all the important effects of insect-like wing kinematics, such as wing rotation and wake capture, which are not included in studies involving unidirectionally revolving wings. When considering reciprocating wings that do include these effects of wing rotation and wake capture, there appears to be only a single study that explores petiolation effects [21]. The aim of this work is to add experimental data and analysis of a reciprocating wing with finer changes in petiolation across the range *P* = 1–3. In particular, petiolation effects on the LEV formation and characteristics will be described. As a reciprocating wing is used in this study, the aforementioned effects from wing rotation and wake capture are inherently included.

## 3. Material and methods

### 3.1. Test wings and kinematics

**Figure 1.**
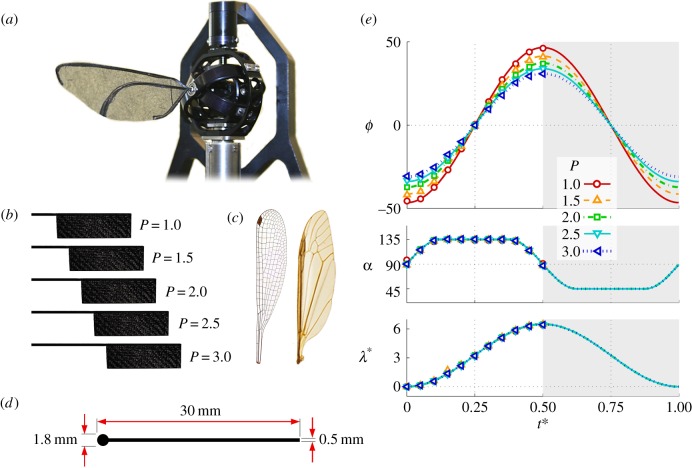
(*a*) Flapperatus; (*b*) tested wings of varying petiolation; (*c*) damselfly (left) and cranefly (right) wings; (*d*) wing cross-section; (*e*) flapping kinematics throughout flapping cycle for each petiolation; lines represent measured mechanism output kinematics; symbols represent measured wing position including any wing flexion at the 11 flow field measurement instances; white and grey regions denote separate half-strokes.

Experiments were performed with a mechanical flapping-wing apparatus known as the ‘Flapperatus’ (figure 1*a*) which enables arbitrary three-dimensional wing kinematics to be produced up to a 20 Hz flapping frequency in air. Details of the design and capabilities are described in detail elsewhere [39,40]. The Flapperatus was used to drive a suite of rectangular wing designs varying in petiolation, illustrated in figure 1*b*. The test wings cover the range *P* = 1–3, with a root-to-tip length (*R*) of 90 mm, and a chord length (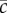) of 30 mm, giving an aspect ratio AR of 3 (defined here for the single-wing as 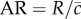). These wings represent a range in Rossby number *Ro* of 4–6 using the definition in [21] based on the ratio of the tip radius *r*_tip_ to the mean wing chord. Owing to the choice in kinematics, Rossby numbers calculated using the original definition with the mean wing angular velocity 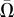 and mean wingtip velocity taken as the characteristic velocities (
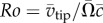
), yield the same values of *Ro* = 4–6. The wings were designed to be stiff to remove effects due to flexibility, and were comprised of a 1 mm diameter carbon fibre rod for the main spar that is sandwiched between two layers of carbon fibre cloth impregnated with resin giving a membrane thickness of 0.5 mm and leading-edge diameter of 1.8 mm (figure 1*d*).

The wings were flapped according to the kinematics illustrated in figure 1*e*, which shows the wing stroke angle *ϕ*, pitch angle *α* and the non-dimensionalized stroke position λ* versus time t*, normalized by the flapping period *T*. Here, λ* is defined as the number of mean chords traversed by the wingtip from the start of the wing stroke. A flat wingtip trajectory was employed with the out-of-plane plunge angle *θ* held at 0°. The lines represent the commanded kinematics by the Flapperatus, and the symbols indicate the actual kinematics including wing flexion at the 11 measurement instances spaced evenly throughout the wing stroke encompassing one half of the flapping cycle. For the out-of-plane angle not shown, *θ* was within the range of ±0.6° throughout all cases. Kinematics were selected to maintain constant conditions across the test cases so as to isolate effects due to petiolation alone. This includes both a constant flapping frequency *f* of 1.8 Hz, which gives an insect-relevant Reynolds number *Re* of 1400. Here, *Re* is based on the mean wing chord, constant mean wingtip speed 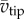 of 0.7 m s^−1^, and the kinematic viscosity *v* (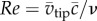). The mean wingtip speed was held constant in an effort to maintain similar wing tip effects through a constant tip vortex strength, following [41]. In addition, the non-dimensional stroke amplitude Λ*, defined as the number of mean wing chords traversed by the wingtip over a wing stroke, was held constant at a value of 6.5 across the cases, typical of insects. This resulted in the wing planforms travelling approximately the same distances, which is an important parameter to maintain as the extent of LEV development has been shown to be strongly dependent on the distance travelled [42,43]. As these kinematics maintain identical wingtip kinematics, the Euler fluid forces at the wing tip, resulting from wing acceleration, also remain constant.

### 3.2. Flow field measurement and analysis

**Figure 2.**
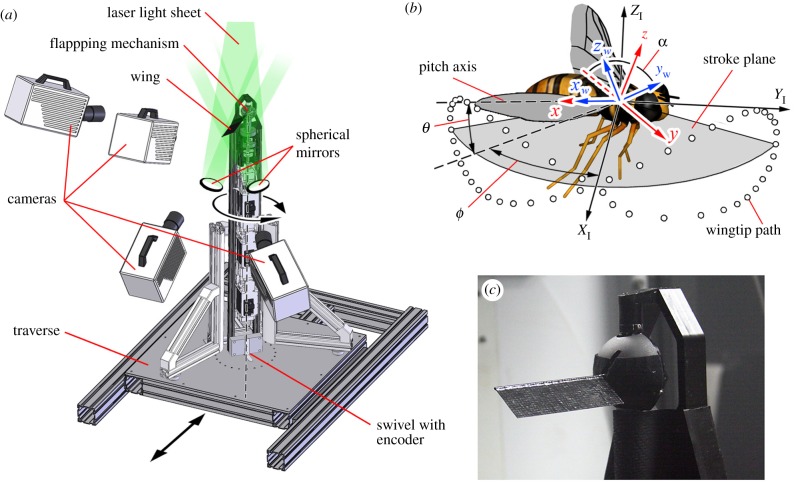
(*a*) Stereo PIV measurement set-up; (*b*) coordinate system definitions; (*c*) close-up of wing mounted to Flapperatus.

Flow field measurements were accomplished using the technique of stereoscopic particle image velocimetry (PIV) employing a set of 1024 × 1024 px high-speed cameras (Photron SA3, Photron Ltd) and a 527 nm 1 kHz Nd:YLF laser (Litron LDY-300PIV, Litron Lasers Ltd, UK) with light sheet optics. The experimental set-up is illustrated in figure 2*a* where four cameras fitted with Scheimpflug lens mounts [44] were arranged to capture data both above and below the wing. Comprehensive illumination was ensured by using mirrors to reflect the light sheet back into the shadow cast by the wing. The upper and lower cameras were fitted with 105 mm lenses (AF Nikkor, f#2.8) and 180 mm lenses (AF Nikkor, f#3.5), respectively. The entire Flapperatus was mounted on a swivel with a rotary encoder and motorized traverse, which enabled phase-locked PIV measurements at a desired point in the flapping cycle to be acquired across the entire wing span, resulting in pseudo-volumetric flow fields. For each of the 11 measurement instances evenly spaced throughout the wing stroke (shown by the symbols in figure 1*e*), flow field measurements were acquired every 1 mm along the span from 2 mm inboard of the root to 15 mm beyond the wingtip and repeated three times. It is emphasized here that the flow measurement instances encompass both the wing rotation (pronation and supination) and translation (downstroke) phases of the flapping cycle for a fully developed flow field. Flow seeding was provided by 1 µm diameter olive oil droplets from an aerosol generator.

Stereo calibration of the measurement area was performed with a dual-plane 105 × 105 mm calibration plate enabling the raw image pairs to be processed into three-component vector maps using DaVis 8.0.8 (LaVision UK Ltd, Oxfordshire). Here, a stereo cross-correlation algorithm was used with an initial interrogation window size of 64 × 64 px progressing to a final window size of 16 × 16 px with a 50% overlap. Further details on the PIV processing settings can be found in [45]. The resulting vector maps for each spanwise measurement location at a given instant were ensemble-averaged and arranged into three-dimensional volumes.

Vortices within the three-dimensional flow field were identified using the objective, automated technique in [46], which finds and classifies critical points, such as foci and saddles, from zero-crossing points in a progressive, step-wise sweep through the volume following the criteria proposed in [47]. Once the vortex core centres have been located, the vortex diameter *D* at any spanwise position can be determined from the width of the solid body rotation region in the local Rankine vortex velocity profile. Circulation *Γ* can be then calculated from the tangential velocity *v*_t_ at the edge of this region (where *Γ* = *πDv*_t_). Three-dimensional vortex axes were determined using the technique described in [39,45] which exploits the fact that the local vorticity vector is tangential to the path of the vortex axis. Flows at the wing surface were visualized using vector maps comprised of velocity measurements located 1 mm from the upper wing surface, and instantaneous streamlines for these and other vector maps were produced using line integral convolution (LIC) [48]. For further details on the methods used for vortex axis identification, vortex diameter and circulation calculation, and near-surface flow extraction, the reader is referred to [45].

We define a set of coordinate systems in figure 2*b*. The inertial ‘*I*’ coordinate system is aligned with the *X_I_*, *Y_I_* and *Z_I_* axes pointing in the lateral, forward, and vertical directions respectively. In the *xyz* coordinate system, the *x* axis is aligned with the wing pitch axis pointing towards the wingtip, the *y* axis lies within the *X_I_Y_I_* plane, and *z* is perpendicular to *x* and *y*. The kinematic patterns used here have negligible plunging motion (*θ*), thus the wing pitch axis always lies in the stroke plane (*X_I_Y_I_*). Lastly, the wing-fixed coordinate system ‘*w*’ consists of the *x_w_*, *y_w_* and *z_w_* axes pointing in the spanwise, chordwise and wing-normal directions respectively.

## 4. Results and discussion

The impacts of petiolation on the LEV and flow development at key spanwise locations will be presented first. Next, the effects on the near-surface flow and three-dimensional flow topology will be presented along with an examination of LEV stability. The effect of petiolation on the LEV lift contribution will then be given, followed by an exploration of the influence of three-dimensional effects on the local flow evolution.

### 4.1. Chordwise planes

**Figure 3.**
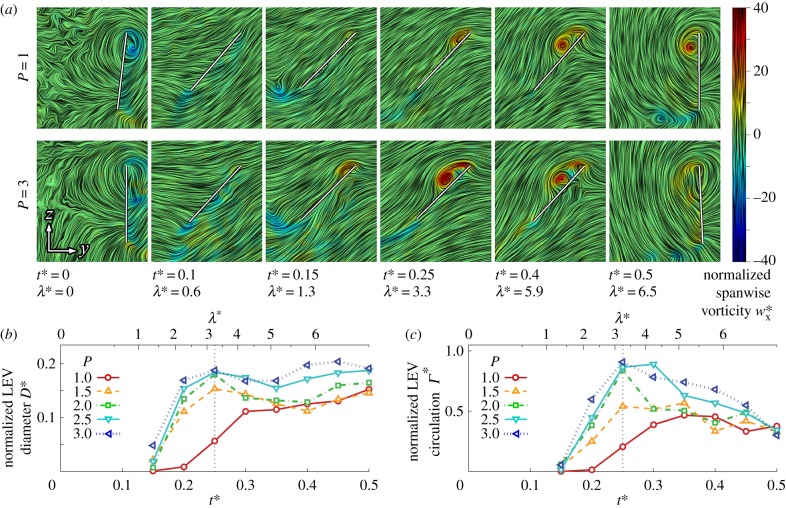
LEV development at 25% span: (*a*) selected chordwise planes of instantaneous streamlines for *P* = 1 and 3; wing chord is denoted by white line; (*b*) normalized LEV diameter D*; and (*c*) normalized LEV circulation Γ* throughout stroke where dashed line denotes mid-stroke.

**Figure 4.**
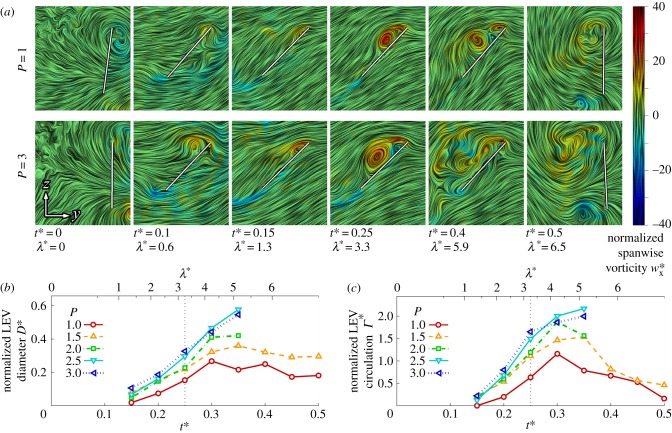
LEV development at 50% span: (*a*) selected chordwise planes of instantaneous streamlines for *P* = 1 and 3; wing chord is denoted by white line; (*b*) normalized LEV diameter D*; and (*c*) normalized LEV circulation Γ* throughout stroke where dashed line denotes mid-stroke.

**Figure 5.**
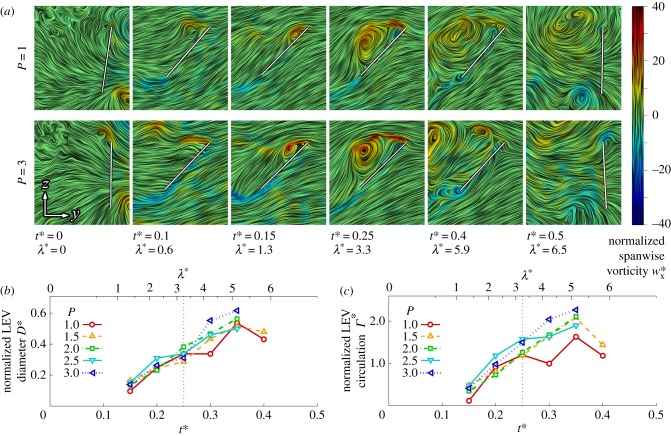
LEV development at 75% span: (*a*) selected chordwise planes of instantaneous streamlines for *P* = 1 and 3; wing chord is denoted by white line; (*b*) normalized LEV diameter D*; and (*c*) normalized LEV circulation Γ* throughout stroke where dashed line denotes mid-stroke.

The flow evolution and LEV development throughout the wing stroke versus petiolation is illustrated in figures 3–5 for 25, 50 and 75% span, respectively. Chordwise planes for the two extreme petiolation cases (*P* = 1 and 3) are given with instantaneous streamlines coloured with normalized (spanwise) vorticity 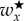 (where 
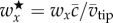
) along with plots of normalized LEV diameter D* (non-dimensionalized by 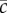), and circulation strength *Γ* * (where 
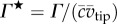
). The same chordwise planes for all measurement instances for *P* = 1, 2 and 3 can be found in the electronic supplementary material.

As seen in the chordwise planes in figures 3*a*–5*a*, flow development across the span is broadly similar from *P* = 1–3. The development is characterized chiefly by the formation of a primary LEV with increasing size and strength towards the wing tip, and which increases in size and strength throughout the wing stroke. Despite this general qualitative similarity, clear effects on the LEV development are evident when examining figures 3*b*,*c*–5*b*,*c*. First, inboard at 25% span it can be seen that the LEV grows in both size and strength more rapidly and reaches greater peak values for a more petiolate wing (figure 3*b,c*). The difference is particularly notable for *P* > 1 in the first half of the wing stroke (t* = 0.25). This is also reflected in the chordwise plots when comparing the LEV size between *P* = 1 and 3 at t* = 0.15–0.25 in figure 3*a*, where the LEV size is substantially larger for the more petiolate wing. After mid-stroke the LEV diameter levels out in all cases and appears to reach a stable size with more petiolate wings exhibiting greater LEV diameters. The LEV strength is also generally higher throughout the second half of the wing stroke for more petiolate wings, with the vortex strength reaching a plateau around mid-stroke for *P* ≤ 1.5, but declining in strength for *P* > 1.5. Over the range tested, the LEV remains attached to the upper wing surface at this inboard region of the wing throughout the entire stroke owing to its restricted growth up to a maximum size of approximately 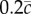.

In contrast with the inboard sections of the wings, at the mid-span position, the LEV growth rates are more comparable across *P* = 1–3 (figure 4*b*,*c*). However, the vortex is generally larger and stronger for more petiolate wings. This is also seen in the chordwise planes in figure 4*a*, when comparing, for instance, the LEV size between *P* = 1 and 3 at t* = 0.25, where the LEV is significantly larger for *P* = 3. The LEV size remains constant after mid-stroke for *P* ≤ 1.5, although this is accompanied by a decline in vortex strength. The higher petiolation cases (*P* > 1.5), however, peak in both size and strength at t* = 0.35, shortly after mid-stroke, after which the LEV shows signs of breakdown and detachment. Evidence of detachment can be seen at t* = 0.4 in figure 4*a* where for *P* = 3 a large recirculation region appears to encompass the entire wing chord with no clear flow reattachment point on the wing surface. This is accompanied by a region of negative spanwise vorticity at the trailing edge, suggesting the formation of a trailing-edge vortex (TEV). It has been reported that LEV detachment is accompanied by the formation of a TEV as the flow reattachment line (aft of the LEV core) falls off the wing trailing edge [24,49]. In comparison, at this instant at t* = 0.4, the LEV remains coherent for the least petiolate wing *P* = 1; there is a smaller recirculation region over the wing surface, a clear flow reattachment point approximately two thirds of the wing chord from the leading edge, and the absence of negative spanwise vorticity at the trailing edge. Thus, in the mid-span region of the wing, the LEV remains attached throughout the wing stroke for the least petiolate wing but as petiolation increases the LEV detaches after mid-stroke.

Finally, in the outboard region of the wing at 75% span, both the LEV diameter and circulation trends throughout the wing stroke for all petiolation cases are very similar (figure 5*b*,*c*). LEV diameter across the cases is comparable (figure 5*b*), with the LEV growing throughout the wing stroke up to a peak around t* = 0.35. The same trend is true for LEV strength; however, in the second half of the wing stroke the LEV appears slightly stronger for the most petiolate wing in comparison with the least petiolate wing. The chordwise plots in figure 5*a* reflect the same trends, where at mid-stroke (t* = 0.25) the flow fields are qualitatively indistinguishable between *P* = 1 and 3. In all cases, the LEV becomes less coherent and shows similar signs of detachment from t* = 0.35 onwards, as was observed at mid-span. However, in contrast with mid-span, the outboard region shows indications of LEV detachment even for the least petiolate wing. Examining t* = 0.4 in figure 5*a*, it can be seen that for both *P* = 1 and 3 that a recirculation region has enveloped the entire wing chord with an associated concentrated region of negative vorticity at the trailing edge. For the most petiolate wing *P* = 3, however, the primary LEV has shed much further away from the wing surface. In general, for this outboard region, the LEV does not remain attached to any of the wing designs throughout the stroke, but instead detaches shortly after mid-stroke.

### 4.2. Near-surface flow

**Figure 6.**
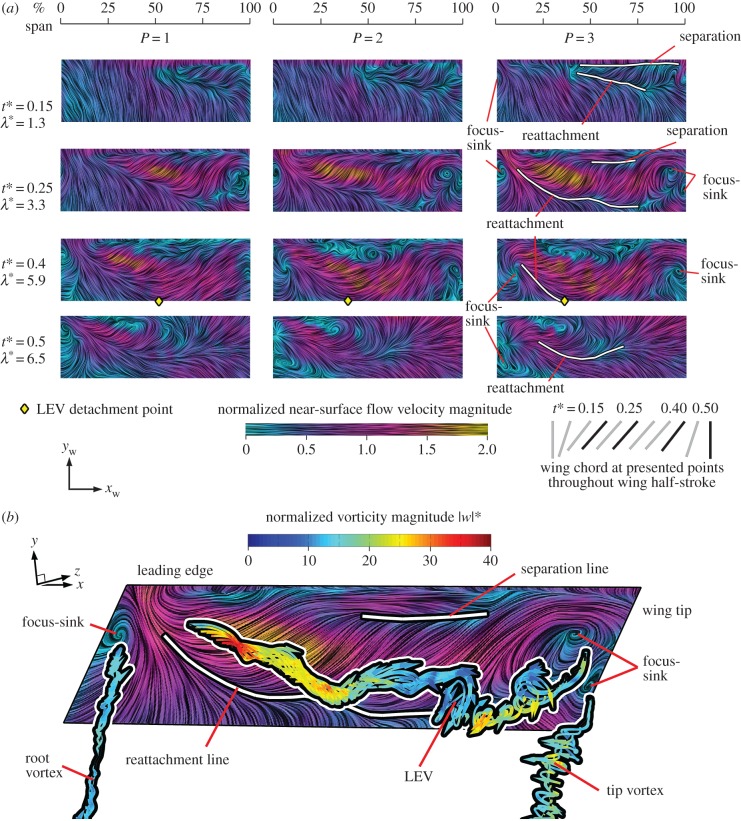
Three-dimensional flow topology: (*a*) selected planform views for *P* = 1, 2 and 3 wing upper surface with near-surface skin friction lines coloured by in-plane normalized velocity magnitude. For each planform, view the leading edge is the top and the wingtip is to the right; (*b*) near-surface skin friction lines on upper wing surface with major vortex structures highlighted by superimposed streamlines coloured by normalized vorticity magnitude for *P* = 3 at mid-stroke.

We now progress to examining the three-dimensional LEV structure and flow topology. This will be accomplished through visualizations of near-surface skin friction lines, coloured with in-plane velocity magnitude normalized by 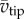 (figure 6). Previously established separation patterns obtained from local solutions to the Navier–Stokes equations, including critical points [47,50,51], can be used to describe and discern near-surface flow patterns. Such an approach has been adopted previously in describing insect flow topologies [8,11,52], and in experimental flapping-wing models [45,49].

Normal views of the upper wing surfaces with near-surface skin friction lines developing throughout the wing stroke are presented for each of the petiolation cases in figure 6*a*. The general flow pattern across the petiolation cases is qualitatively similar so, for clarity, approximate reattachment and separation lines and focus-sink critical points are given for *P* = 3 only. Shortly after the beginning of the wing stroke (t* = 0.15), when the wing has reached a steady angle of attack and is still accelerating, the near-surface flow is very similar across all cases. At this early stage, the flow is largely attached and oriented in the chordwise direction towards the trailing edge. A distinct triangular region of reverse flow is present near the wing tip close to the leading edge. This region is bounded by separation and reattachment lines. This is the ‘footprint’ of the LEV over the wing surface, providing an indication of the extent of its shape and size. The LEV appears to extend further inboard for the most petiolate wing. In each case, the LEV probably originates from an open negative bifurcation line type separation, which is one of the fundamental separation types described in [50] where the surface flow converges to a separation line. In this case, the separation line feeding the LEV lies, as expected, along the leading edge.

Progressing to mid-stroke (t* = 0.25), when the wing has reached its peak velocity, the aft reattachment line extends inboard and differences in the flow topography with varying degrees of petiolation begin to emerge. The LEV footprint is distinctly larger for the more petiolate wings. Furthermore, the near-surface flow velocities are notably higher within this region. These two features together suggest that the LEV is both larger and stronger for higher wing petiolation, particularly in the mid-span and inboard locations. This is consistent with the observations made in §4.1 where LEV diameter and circulation values were shown to be higher for greater *P*. A larger footprint area on the wing surface combined with a stronger vortex (given by the tangential flow speed around the core) would be expected to generate greater lift in more petiolate wings. This will be assessed in more detail in the next section. The change in the LEV footprint shape with *P* also suggests that the LEV becomes more cylindrical in shape with increasing petiolation, rather than conical. For *P* = 1, the LEV footprint is distinctly triangular, whereas for *P* = 3, the footprint width is more consistent along the wingspan. The velocity distribution along the span becomes more constant with increasing petiolation, and is the likely cause of a more constant LEV diameter along the span. The separated LEV flow again probably originates from an open negative bifurcation line, whereas the tip and root vortices likely originate from a different separation type.

To help illustrate the tip and root vortex separations in detail, figure 6*b* presents the near-surface flow pattern with instantaneous streamlines released from axes of the major vortex structures (primary LEV, tip and root vortex) coloured with normalized vorticity magnitude |*w*|* (where 
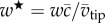
) for *P* = 3 at mid-stroke. Here, it can be seen that the tip and root vortices begin in the vicinity of a focus-sink on the wing surface. This is known as a Werlé–Legendre separation [50] in which the surface flow converges to a focus plus sink and leaves the surface where a vortex axis is anchored at the centre of the focus. In addition, the outboard end of the LEV appears to originate from a second focus-sink on the wing surface, thus, the LEV is fed by both an open negative bifurcation line and Werlé–Legendre separations. Similar surface flow features and topologies are present for all the petiolation cases tested. As a result of the LEV being anchored at its tip-ward end, the LEV appears arch-shaped. This LEV form is also observed for the other petiolation cases, where it is consistently seen to be arch-like at its outboard end. An arched-LEV of this kind on a revolving wing has been reported elsewhere [41,45,53,54], and most relevant to this study, in [30]. In addition, studies on translating wings have also observed an arch-shaped LEV [24,55]. In the studies of [30], whose two experimental test cases of a revolving AR = 2 wing represent *P* = 0.63 and 3.62, revealed two distinct classes of LEVs. These included a conical and arch-shaped LEV for the lower and higher petiolation cases respectively. This, combined with our results, suggest that the LEV transitions from a conical form towards a more arch-like form somewhere in the range of *P* = 0.63–1 and also at variable times in the stroke cycle. The LEV form will have implications for the planform shape requirements, where the wing should be designed such that the LEV size does not exceed the local chord length anywhere along the span, otherwise LEV detachment is likely to occur. With this in mind, a conical LEV would allow for a wing planform that decreases in chord length towards the root, whereas an arched-LEV, more consistent in diameter, would require a comparatively more constant chord length along the span. The latter case requires more wing area inboard, shifting the centre of pressure location closer to the root.

The arch-like form of the LEV suggests that at this stage in the wing stroke the LEV and tip vortex are not one continuous structure, because the outboard end of the LEV terminates on the wing surface. Visualizations of vortex cores in the work of [54] show a similar feature part way through the wing stroke where there is a disconnect (or ‘kink’) between the outboard arched-LEV and the tip vortex, resembling two separate structures. It is possible that a non-continuous LEV and tip vortex system is a result of wingtip geometry, as the present, and past studies reporting an arched-LEV [41,45,53,54] all employ a rectangular revolving wing. A similar picture of separate arched-LEV and tip vortex structures was shown in [55] for a translating (heaving) rectangular wing. It was observed that the LEV was ‘pinned’ to the wing front corners as a consequence of the tip vortices which induce a flow pushing the outboard portions of the LEV down to the wing surface. Thus, the arched-LEV structure observed in this work is probably due in part to the influence of the tip vortex.

**Figure 7.**
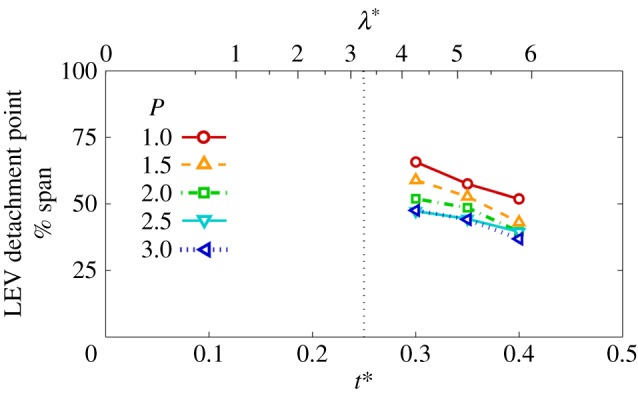
LEV detachment point along span throughout stroke versus *P*; detachment point is taken as the point where flow reverses at the trailing edge.

As the wing begins to decelerate after mid-stroke (t* = 0.4), the LEV footprint continues to grow in size in all cases (figure 6*a*). Similar to mid-stroke, the tip and root vortices are again observed to originate from Werlé–Legendre type separations. Despite the growth in the footprint size, the near-surface flow velocities in the LEV region have diminished. This is probably due, in part, to the fact that the LEV is seen to detach from the wing outboard at this instant for all petiolation cases. The spanwise detachment point can be objectively identified by locating the point at the trailing edge where the near-surface flow reverses direction. As noted previously (§4.1), LEV detachment occurs when the aft end of the LEV reaches the trailing edge, initiating reversed flow and the formation of a TEV [24,49]. Applying this criterion to all cases, the detachment point along the wingspan is plotted for each petiolation case throughout the wing stroke in figure 7. Here, it can be seen that detachment initiates shortly after mid-stroke and then progresses inboard at similar rates for all petiolation cases. A trend is clearly seen where, for any given instant, LEV detachment occurs further inboard for a more petiolate wing. For instance, at t* = 0.3 the LEV detaches at approximately 70% span for *P* = 1, whereas for *P* = 3 it detaches significantly further inboard at roughly 50% span. This outcome that the LEV on a more petiolate wing is less stable and more prone to detachment is to be expected because increasing petiolation effectively increases local Rossby number for a given per cent span, and the Rossby number has been strongly linked to LEV stability [21]. Returning to figure 6*a*, beyond t* = 0.4 (as the wing pitches up rapidly), the flow at the trailing edge along the entire wing length in each case is restored to a slow, common aftward direction as the TEV is shed into the wake and the reattachment line shifts forwards towards the leading edge (t* = 0.5). At this point in the cycle, the outboard focus-sink points on the wing surface previously seen for the outboard LEV and tip vortex origins have disappeared, suggesting that the outboard LEV and tip vortex have transitioned to a continuous structure. This is consistent with observations made in [54] who reported that the LEV is initially arch-shaped, but then lifts off the outer wing surface and reorients itself with the tip vortex.

### 4.3. Leading-edge vortex lift

**Figure 8.**
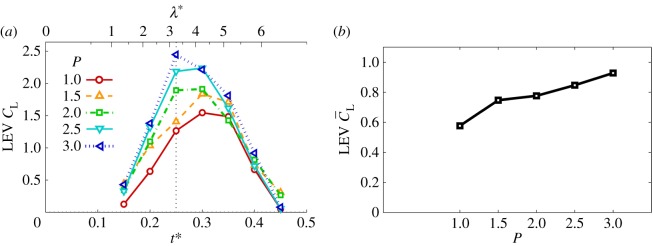
(*a*) LEV circulatory lift coefficient throughout half-stroke for varying *P*; (*b*) time-averaged LEV circulatory lift coefficient versus *P*.

With the LEV geometry identified in each case, the local LEV lift across the span can be determined from the circulation values in combination with the local instantaneous wing speed according to the Kutta–Joukowski theorem. The resulting lift can then be integrated along the span to obtain a value for the overall contribution from the LEV. It has been shown in [56] that the majority of the total lift arises from circulation contained in the LEV, rather than bound circulation. This was concluded from an experimental study employing a translating wing paired with a two-dimensional potential flow model not encompassing three-dimensional effects. Despite notable kinematic differences between their study and ours, they both concern separated flows featuring an LEV. Accordingly, their finding would suggest that the LEV is also the major contributor to the overall lift here. Figure 8*a* presents LEV lift coefficients for each instant in the wing stroke for all petiolation cases. Here, it can be seen that lift coefficients are generally higher for more petiolate wings. In the first half of the wing stroke, lift values are especially higher, which is probably due to the fact that over this period the LEV circulation values inboard and at mid-span were seen in §4.1 to be higher for larger values of *P*, and the LEV footprint on the wing surface was also larger (§4.2). Beyond mid-stroke, lift coefficient values decline with the wing speed and are more comparable in magnitude across the petiolation cases. This probably occurs due to LEV detachment occurring further inboard for higher *P* at a given instant in this phase of the cycle, resulting in lift reduction for more petiolate wings. Thus, it appears that over the first half of the wing stroke, more petiolate wings benefit from enhanced lift originating from a larger and stronger LEV across the span, but then suffer from comparatively greater reductions in lift in the second half of the wing stroke when LEV detachment is more pronounced.

By time-averaging the trends in figure 8*a*, the mean LEV lift values over the wing stroke are plotted against petiolation in figure 8*b*. It can be seen that, over the range tested, increasing petiolation leads to larger mean LEV lift. Again, this is because the LEV is generally larger and stronger across the span. This finding is contrary to those of [21] and [57] who found a decrease in total lift with increasing petiolation. While LEV lift increases with petiolation, it remains possible that when other non-circulatory lift contributions are included, then the net result is a decline in total lift. If so, then further investigation is warranted to determine which kinematic parameters contribute to the overall decline.

Recall from §1 that in the case of a rotating wing with increasing petiolation, the characteristic velocity should either be axis-relative or root-relative, following [28]. The lift coefficients presented here were obtained with 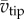 as the characteristic velocity, which is an axis-relative value. For comparison, lift coefficients were recalculated and plotted with a root-relative velocity of the mean wing speed at 75% span, which compensates for the fact that more petiolate wings have a higher average velocity across the wingspan. The resulting trends were found to be less pronounced than shown in figure 8*a*,*b*; however, the same trends remain. With the axis-relative values as given in figure 8*b*, the LEV lift coefficient increases by approximately 61% from *P* = 1–3, whereas with root-relative values the increase is 39%.

### 4.4. Implications of petiolation for flappercraft

In addition to a rise in LEV lift, greater petiolation may offer increased control authority. With the wing area located further outboard, manoeuvring torques around all three axes will be higher because of the longer moment arms for aerodynamic forces. Unfortunately, the benefits of greater torques will be offset by increased moments of inertia and damping from added mass as petiolation increases. The net effect of these factors could result in a more petiolate wing giving improved roll, pitch and yaw control authority but this will depend heavily on the wing planform and mass distribution. Increasing petiolation will be associated with an increased power requirement to generate the torques necessary for flapping because the wing mass and centre of pressure both move distally. As the resultant aerodynamic force acts nearly perpendicular to the wing surface, the increase in LEV lift with *P* found in this study will be accompanied by a proportionate increase in drag. For this reason, increases in petiolation will incur an additional penalty of increased power requirements due to drag. This may be offset, however, by reducing flapping frequency because, for a given vehicle mass, flapping frequency can be reduced as petiolation (and hence lift) increases in order to maintain a constant mean lift output.

Genetic manipulations of fruit fly wing planforms have shown increased flight agility when reducing the moment of inertia by moving wing area inboard while simultaneously maintaining the position of the centre of pressure [58]. However, flight efficiency decreased, and it will be reduced further for petiolate wings because, as the contralateral wings become more aerodynamically independent, span efficiency is likely to decrease accordingly. This appears to be the case of a bumblebee operating with aerodynamically independent wings, versus a flier with a more even downwash and greater span efficiency, such as a desert locust [52,59]. Furthermore, span efficiency has been shown to vary directly with wing taper ratio, where damselflies with low taper ratio (wing area concentrated distally) petiolate wings have worse performance than high taper ratio non-petiolate dragonfly wings [31].

Beyond aerodynamic reasons, stresses at the wing base will also increase with petiolation and drag-induced torques. It is quite possible that petiolate wings may be aerodynamically beneficial under certain circumstances, but may not be attractive for flappercraft and are not common in the natural world due to intolerable power requirements or the robustness of the wing bases to higher stresses.

### 4.5. Three-dimensional effects

**Figure 9.**
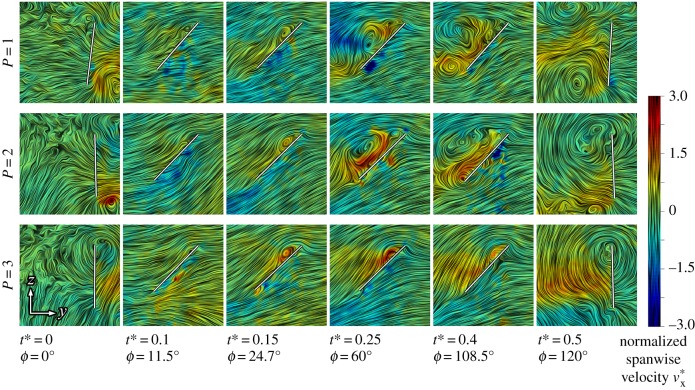
Comparable chordwise planes of instantaneous streamlines for *P* = 1, 2 and 3 at r* = 3.5 with identical flapping kinematics; wing chord is denoted by white line.

We now shift focus to investigating the influence of three-dimensional effects on the flow development through varying wing petiolation. For this purpose, flow field measurements were performed for a separate set of kinematics in which each wing petiolation case was flapped with identical kinematics employing a 120° stroke amplitude. The wing stroke kinematics remained sinusoidal, along with identical pitching kinematics presented in figure 1*e*, and a constant flapping frequency of 1.4 Hz. As stroke amplitude and flapping frequency were held constant while petiolation increased, this resulted in a variation in Reynolds number from 1400 for *P* = 1 up to 2100 for *P* = 3. Chordwise planes at a set normalized radial (rather than spanwise) position r* of 3.5 (normalized by 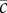) were then examined. The result of this is presented in figure 9 illustrating these constant planes throughout the wing stroke for *P* = 1–3, with instantaneous streamlines coloured by normalized spanwise flow 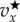 (normalized by the local mean wing velocity of 0.61 m s^−1^ at this radial position). As kinematics are held constant across all cases, the presented chordwise planes experience identical local conditions including constant local wing speeds, chords travelled (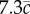), and identical centrifugal and Euler fluid forces. If the flow were predominately two dimensional and the LEV development were only influenced by shed vorticity from the local leading edge and viscous dissipation with no spanwise vorticity transport, then all of the planes presented in figure 9 would be identical. However, this is clearly not the case as the flow development varies dramatically as petiolation changes. These differences are not due to variation in Reynolds number because it has been shown that Reynolds number has little effect on LEV development and general structure over the range 200 ≤ *Re* ≤ 60 000 [34], which extends far beyond the range covered by the present test cases.

For *P* = 3, the set radial plane corresponds to the inboard region of the wing at approximately 17% span, and it can be seen that a small LEV forms and remains attached to the wing for the entire duration of the wing stroke. By contrast, for *P* = 1 and 2 with planes corresponding to 83 and 50% span respectively, the pattern is very different. Initially the LEV is a comparable size across all the cases up to t* = 0.15, after which the LEV grows dramatically by mid-stroke and exhibits signs of detachment further on at t* = 0.4 when the recirculation region envelops the entire wing chord and a TEV has formed. Returning to *P* = 3, a strong region of positive spanwise flow (directed towards the wingtip) is concentrated and maintained in the core of the LEV throughout. With this in mind, it makes sense that the LEV remains a stable size, as a strong spanwise flow will transport vorticity generated at that local wing chord away to more outboard locations rather than allowing it to build up locally leading to vortex growth. Furthermore, Coriolis forces, which have been linked to LEV stability [21], are proportional to the spanwise flow velocity. Thus, we can expect that a local LEV with a strong spanwise flow would remain attached to the wing because it will be accompanied by stronger stabilizing Coriolis forces. By contrast, *P* = 1 and 2 representing the outboard and mid-span locations, respectively, have comparatively slower spanwise flows along their LEV cores. As a result, it appears that locally generated vorticity is augmented by vorticity arriving at this radial position from more inboard locales, leading to a large rise in LEV diameter and eventual detachment. As the local conditions at these wing chords are identical—essentially they would be identical elements of a blade element model—any differences in the local flows must originate from three-dimensional effects: namely, adjacent sections of the LEV, the influence of the tip vortex, and pressure forces arising from the gradient in the local wing velocity along the span.

## 5. Conclusions

The effect of wing petiolation on the three-dimensional flow field and LEV development was investigated experimentally on an AR 3 rectangular wing, over the petiolation range *P* = 1–3. Kinematics were selected such that a constant Reynolds number, mean number of chords travelled by the wingtip, and flapping frequency were maintained across the test cases so that effects due to varying petiolation could be isolated and tested. Increasing petiolation was found to lead to a general rise in LEV size and strength, particularly at mid-span and inboard regions. In the first half of the wing stroke, the LEV is notably larger and stronger as these quantities grow more rapidly and reach higher peak values for more petiolate wings. Over the second half of the wing stroke, however, the LEV detaches further inboard for higher wing petiolation, commencing shortly after mid-stroke at approximately 70 and 50% span, respectively, for *P* = 1 and 3 and progressing further inboard thereafter. Flow development at a given radial position was shown to be heavily influenced by three-dimensional effects, most likely from the tip vortex and spanwise pressure gradients. The increase in LEV diameter and strength with petiolation was found to be accompanied by an increase in LEV footprint size on the upper wing surface, where the most dramatic changes, again, were seen mid-span and inboard. The three-dimensional LEV initially takes on an arch-like form in each case, and is fed by an open negative bifurcation line type separation and a Werlé–Legendre separation at its outboard end where it is anchored to the wing surface. Owing to this form, the LEV appears as a separate structure from the tip vortex. Tip and root vortices were observed to originate from Werlé–Legendre separations in each case. Towards the end of the wing stroke, the LEV and tip vortex transition to one continuous structure as outboard separation points disappear from the surface flow pattern. As a consequence of a larger and stronger LEV occupying a bigger surface footprint, higher wing petiolation results in higher LEV lift coefficients, especially in the first half of the wing stroke. Coefficients then become more comparable in the second half of the wing stroke, probably due to the negative effects of LEV detachment on more petiolate wings. The net result is a general rise in the mean LEV lift coefficient with petiolation. In the context of robotic flappercraft, our results suggest that petiolate wings may produce more total lift, but would come at a cost of reduced flight efficiency and greater power required for flight due to higher drag-induced torques. This, in turn, indicates that petiolate wings could be suitable for flappercraft that prioritize high payload capacity or high-acceleration manoeuvres over flight efficiency.

## Data accessibility

The datasets supporting this article have been uploaded to the Dryad Digital Repository at http://dx.doi.org/10.5061/dryad.jv280.
